# Strategic Response to an Outbreak of Circulating Vaccine-Derived Poliovirus Type 2 — Syria, 2017–2018

**DOI:** 10.15585/mmwr.mm6724a5

**Published:** 2018-06-22

**Authors:** Chukwuma Mbaeyi, Zubair Mufti Wadood, Thomas Moran, MJourn, Fazal Ather, Tasha Stehling-Ariza, Joanna Nikulin, Mohammad Al Safadi, Jane Iber, Laurel Zomahoun, Nidal Abourshaid, Hong Pang, Nikki Collins, Humayun Asghar, Obaid ul Islam Butt, Cara C. Burns, Derek Ehrhardt, Magdi Sharaf

**Affiliations:** ^1^Global Immunization Division, Center for Global Health, CDC; ^2^Polio Eradication Department, World Health Organization, Geneva, Switzerland; ^3^Middle East and North Africa Office, United Nations Children’s Fund, Amman, Jordan; ^4^Division of Global Health Protection, Center for Global Health, CDC; ^5^Division of Viral Diseases, National Center for Immunization and Respiratory Diseases, CDC; ^6^Syria Country Office, United Nations Children’s Fund, Damascus, Syria.

Since the 1988 inception of the Global Polio Eradication Initiative (GPEI), progress toward interruption of wild poliovirus (WPV) transmission has occurred mostly through extensive use of oral poliovirus vaccine (OPV) in mass vaccination campaigns and through routine immunization services (*1*,*2*). However, because OPV contains live, attenuated virus, it carries the rare risk for reversion to neurovirulence. In areas with very low OPV coverage, prolonged transmission of vaccine-associated viruses can lead to the emergence of vaccine-derived polioviruses (VDPVs), which can cause outbreaks of paralytic poliomyelitis. Although WPV type 2 has not been detected since 1999, and was declared eradicated in 2015,* most VDPV outbreaks have been attributable to VDPV serotype 2 (VDPV2) (*3*,*4*). After the synchronized global switch from trivalent OPV (tOPV) (containing vaccine virus types 1, 2, and 3) to bivalent OPV (bOPV) (types 1 and 3) in April 2016 (*5*), GPEI regards any VDPV2 emergence as a public health emergency (*6*,*7*). During May–June 2017, VDPV2 was isolated from stool specimens from two children with acute flaccid paralysis (AFP) in Deir-ez-Zor governorate, Syria. The first isolate differed from Sabin vaccine virus by 22 nucleotides in the VP1 coding region (903 nucleotides). Genetic sequence analysis linked the two cases, confirming an outbreak of circulating VDPV2 (cVDPV2). Poliovirus surveillance activities were intensified, and three rounds of vaccination campaigns, aimed at children aged <5 years, were conducted using monovalent OPV type 2 (mOPV2). During the outbreak, 74 cVDPV2 cases were identified; the most recent occurred in September 2017. Evidence indicates that enhanced surveillance measures coupled with vaccination activities using mOPV2 have interrupted cVDPV2 transmission in Syria.

## Context for VDPV2 Emergence in Syria

The ongoing civil war in Syria, which began in 2011, has had a deleterious impact on its health care system, leading to a steep decline in routine vaccination coverage and population immunity. Before the war (during 2001–2010), national 3-dose OPV (OPV3) coverage estimates by age 1 year consistently exceeded 80%. By 2016, estimated OPV3 coverage had decreased from 83% in 2010 to 48% (*8*).

Multiple rounds of supplementary immunization activities (SIAs), implemented in response to a WPV outbreak during 2013–2014 (*9*), mitigated the poor quality of routine immunization services in parts of the country; however, the frequency and quality of these activities lessened after the outbreak was declared over. In 2016, the year after the response to the WPV outbreak officially concluded, six SIAs were conducted in Deir-ez-Zor governorate; two used tOPV, with reported administrative coverage^†^ of 7% and 23%.

The vaccination status of patients aged 6–59 months with nonpolio AFP (NPAFP) can be used as a proxy for OPV vaccination coverage. Among children born during the war, the estimated proportion of unvaccinated children with NPAFP increased from 3% in 2015 to 6% nationally by the end of 2016. In Deir-ez-Zor governorate, the proportion of children aged 6–59 months with NPAFP who had not received OPV rose from 0% in 2015 to 10% in 2016, coinciding with a decline in SIA quality in the governorate. Intermittent bans on vaccination campaigns were also imposed by local authorities in control of the governorate during this period.

Detection of poliovirus circulation depends on prompt identification and investigation of AFP cases. During 2016–2017, national NPAFP rates (assessing surveillance sensitivity) and the proportion of adequate stool specimens collected from AFP patients (assessing quality of case investigation) exceeded the performance targets of ≥2 cases per 100,000 persons aged <15 years and ≥80%, respectively. However, subnational AFP surveillance gaps were noted for both indicators; the proportion of AFP cases in Deir-ez-Zor governorate with adequate stool specimens declined from 84% in 2015 to 61% in 2016.

## Outbreak Epidemiology

**FIGURE 1:**
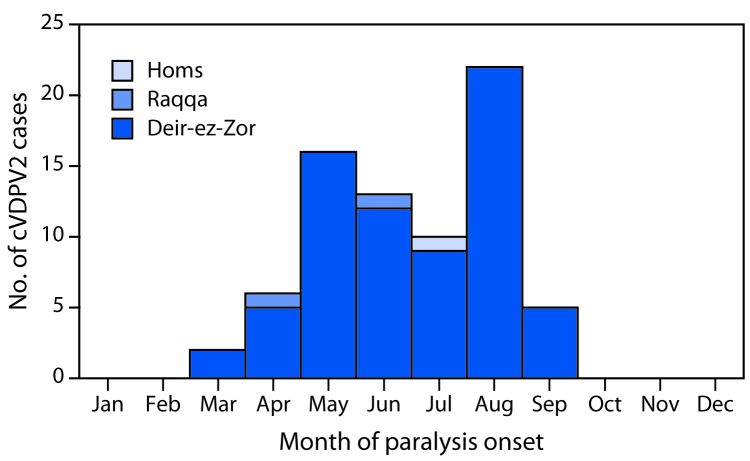
Number of cases of circulating vaccine-derived poliovirus type 2 (cVDPV2), by governorate and month of paralysis onset (n = 74) — Syria, 2017

**FIGURE 2:**
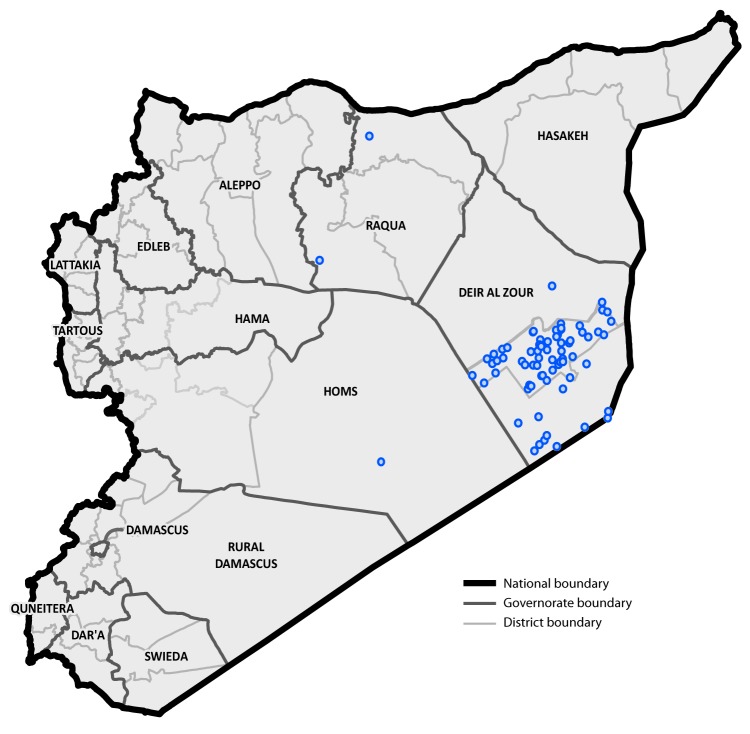
Geographic distribution of cases* (n = 74) of circulating vaccine-derived poliovirus type 2 — Syria, 2017 The figure above is a map showing the geographic distribution of cases (n = 74) of circulating vaccine-derived poliovirus type 2 in Syria during 2017. **Sources:** World Health Organization; Office of Public Health Preparedness and Response, CDC. * Each dot represents one case. Dots are randomly placed within the district boundary.

The earliest identified cVDPV2 outbreak case occurred in a girl aged 22 months from Mayadeen district, Deir-ez-Zor governorate, (paralysis onset March 3, 2017) and the most recent occurred in an infant aged 5 months from Boukamal district, Deir-ez-Zor governorate (paralysis onset September 21, 2017). The cVDPV2 isolate identified in the index case differed from Sabin vaccine virus by 22 nucleotides in the VP1 coding region (903 nucleotides). Among 74 cases reported as of June 17, 2018 (Figure 1) (Figure 2), 46 (62%) occurred in females. The median patient age was 15 months, with 26 (35%) aged <12 months, 35 (47%) aged 12–23 months, 11 (15%) aged 24–59 months, and two (3%) aged ≥5 years. Thirty cases (41%) occurred in children who had never received a dose of OPV, 32 (43%) in 1–2 dose recipients, and 12 (16%) in children who had received ≥3 OPV doses.

Geographically, 71 (96%) cases were reported from Deir-ez-Zor governorate, including 58 (78%) from Mayadeen district, 12 (16%) from Boukamal district, and one (1%) from Deir-ez-Zor district. The three remaining cases were reported (one case each) from Tell Abyad and Thawra districts in Raqqa governorate and Tadmour district in Homs governorate, which borders Mayadeen district, the epicenter of both the current cVDPV2 and the 2013–2014 WPV outbreaks.

## Outbreak Response Activities

Active searches for AFP cases were intensified in districts reporting cVDPV2 cases and in surrounding areas. AFP cases were promptly investigated, and stool specimens were also collected from close patient contacts for testing; the cVDPV2 cases in Raqqa governorate were confirmed through the testing of stool specimens obtained from contacts. In addition, stool specimens were collected from healthy children in affected areas of Deir-ez-Zor governorate and from children arriving in other governorates from outbreak-affected areas. To facilitate early detection of poliovirus circulation, six environmental surveillance sites were established in five governorates (Deir-ez-Zor, Raqqa, Homs, Damascus, and Aleppo) beginning in December 2017. To date, no cVDPV2 has been isolated from sewage samples collected from these sites.

In response to the outbreak, SIAs were implemented in two distinct phases (Table). The first phase took place from July to October 2017, during which time two mOPV2 vaccination campaigns were conducted, targeting children aged <5 years in Deir-ez-Zor and Raqqa governorates. Inactivated poliovirus vaccine (IPV) was also administered to children aged 2–23 months in both governorates during the second mOPV2 round, except in Tell Abyad district of Raqqa governorate. In Deir-ez-Zor governorate, the first mOPV2 vaccination campaign was implemented during July 22–27, 2017. Based on administrative data, approximately 79% of 328,000 targeted children were vaccinated, whereas estimated coverage from postcampaign monitoring data (based on parental recall) was 88%. The second mOPV2 vaccination campaign in Deir-ez-Zor governorate, which was held 1 month later, achieved an estimated 77% administrative and postcampaign monitoring coverage. In Raqqa governorate, the commencement of both mOPV2 campaigns was delayed because of protracted negotiations with multiple local authorities in control of different parts of the governorate. The first mOPV2 vaccination campaign was implemented during August 12–18, 2017. Although administrative data indicated that 86% of 120,000 targeted children were vaccinated, postcampaign monitoring estimated coverage at only 57%. Administrative vaccination coverage was 114% after the second mOPV2 vaccination campaign in Raqqa, implemented in early October 2017; however, estimated coverage from postcampaign monitoring data was 84%.

**TABLE:** Vaccination activities in response to an outbreak of circulating vaccine-derived poliovirus type 2 — Syria, 2017–2018

Governorate/Vaccine type	Target age group (mos)	Outbreak response phase 1				Outbreak response phase 2	
		Round 1		Round 2		Round 3	
		Administrative coverage* (%)	PCM^†^ (recall) (%)	Administrative coverage* (%)	PCM^†^ (recall) (%)	Administrative coverage* (%)	PCM^†^ (recall) (%)
**Deir-ez-Zor**							
mOPV2	<60	79	88	77	77	79	90
IPV	2–23	—	—	71	80	—	—
**Raqqa**							
mOPV2	<60	86	57	114	84	86	84
IPV	2–23	—	—	50	50	—	—
**Hasakeh** ^§^							
mOPV2	<60	—	—	—	—	77	91
IPV	2–23	—	—	—	—	95	85

**Abbreviations:** IPV = inactivated poliovirus vaccine (contains types 1, 2, and 3); mOPV2 = monovalent oral poliovirus vaccine type 2; PCM = postcampaign monitoring.

* Administrative coverage was calculated using denominators for the target age group provided by official sources. These denominators might not have reflected the actual target population because of frequent population movement to and from the outbreak-affected area.

**^†^** Postcampaign monitoring is often considered a more accurate measure of vaccination coverage than administrative data and was estimated using cluster and market surveys administered by independent monitors to parents of children within the target age group.

^§^ Although no case was identified in Hasakeh governorate, it was included in response activities because of its geographic proximity to the outbreak-affected area.

In January 2018, the second phase of response vaccination activities was initiated because of evidence of ongoing cVDPV2 transmission after the first phase of vaccination activities in Deir-ez-Zor governorate. A third mOPV2 vaccination campaign was implemented in Deir-ez-Zor, Raqqa, and Hasakeh governorates as well as in Tadmour district of Homs governorate. IPV was also administered to children aged 2–23 months among groups at high risk in Damascus, Aleppo, and Hasakeh governorates during a separate round of vaccination activities in February 2018. Estimated mOPV2 coverage based on postcampaign monitoring ranged from 84% in Raqqa governorate to 91% in Hasakeh governorate. Fewer than 1,000 children were vaccinated in the sparsely populated district of Tadmour in Homs governorate. Whereas estimating the target population of eligible children in Tadmour district was challenging because of population migration, the number of children vaccinated was seen as reflective of those present in the governorate at the time of the campaign. IPV was also administered to internally displaced children in Syria throughout the second half of 2017, as well as to refugees and groups at high risk in neighboring communities in Lebanon, Iraq, and Turkey, to bolster protection of children in high-risk areas outside the immediate outbreak-affected area.

In line with GPEI protocol for containment of type 2 polioviruses, a principal focus of the response was ensuring proper management and disposal of mOPV2 vaccine vials. Nearly all mOPV2 vaccine vials were successfully retrieved at the end of each round of vaccination activities. Unused vials were returned to national stockpiles, whereas partially used or empty vials were consolidated and destroyed at designated locations. Another crucial element of the response was the tailoring of social mobilization and communication strategies to foster positive perceptions of vaccines while dispelling misconceptions.

## Discussion

A longstanding humanitarian crisis precipitated by war and political unrest has left much of Syria’s population vulnerable to recurrent disease outbreaks (*10*). The 2017 cVDPV2 outbreak followed a WPV outbreak during 2013–2014 and occurred against the backdrop of declining routine vaccination coverage since the onset of the war (*8*) and the poor quality of tOPV SIAs implemented in conflict-affected areas such as Deir-ez-Zor before the 2016 global tOPV-to-bOPV switch.

Given VDPVs’ propensity for emerging in settings of low OPV coverage, the worsening poliovirus type 2 immunity profile among children in Deir-ez-Zor governorate created the conditions for emergence and rapid spread of VDPV2 within the governorate. According to genomic sequence analysis, the viral strain responsible for the outbreak differed by 22 nucleotides from Sabin vaccine virus and was circulating for approximately a year before isolation of VDPV2 in the index case. The delay in detecting circulation of the virus could have contributed to the size and scope of the outbreak, one of the largest documented cVDPV2 outbreaks. Subnational gaps in AFP surveillance performance, as well as delays in receiving laboratory results because of difficulties transporting stool specimens, occasioned by the complex humanitarian emergency, contributed to the inability to detect the outbreak earlier.

Despite immense operational and security constraints, the response by the Syrian national polio eradication program to the outbreak appears to have been effective. Evidence indicates that institution of environmental sewage sampling to supplement intensified AFP surveillance activities and vaccination campaigns with mOPV2 successfully interrupted the transmission of cVDPV2 in Syria.

**Summary**

**What is already known about this topic?**

Oral poliovirus vaccine (OPV) contains live attenuated viruses, which rarely revert to neurovirulence. These vaccine-derived polioviruses (VDPVs) tend to emerge in populations with low OPV coverage and are capable of causing paralysis.

**What is added by this report?**

In 2017, an outbreak of circulating VDPV type 2 (cVDPV2) occurred in Syria, causing 74 cases. Implementation of three rounds of monovalent OPV type 2 campaigns coupled with intensified surveillance interrupted the outbreak.

**What are the implications for public health practice?**

The outbreak in Syria underscores the risk for emergence of vaccine-derived polioviruses in settings of low OPV coverage. High-quality surveillance and targeted vaccination using monovalent OPV type 2 are effective in controlling cVDPV2 outbreaks.

## Footnotes

* http://polioeradication.org/news-post/global-eradication-of-wild-poliovirus-type-2-declared/.

† Administrative vaccination coverage is obtained by dividing the total number of vaccine doses administered by the estimated target population group.
